# Rethinking the interpretation of spring phenological temperature sensitivity

**DOI:** 10.1038/s44383-025-00004-6

**Published:** 2025-08-04

**Authors:** Manuel G. Walde, Yann Vitasse, E. M. Wolkovich

**Affiliations:** 1 Research Unit Forest Dynamics, Swiss Federal Institute for Forest, Snow and Landscape Research WSL, Zürcherstrasse 111, Birmensdorf, 8903 Zürich Switzerland; 2 Oeschger Centre for Climate Change Research, University of Bern, Hochschulstrasse 4, Bern, 3012 Bern Switzerland; 3 Faculty of Forestry, University of British Columbia, 2424 Main Mall, Vancouver, BC V6T 1Z4 British Columbia Canada

**Keywords:** Plant development, Plant ecology, Environmental sciences

## Abstract

Recent publications showed a substantial decline in spring phenological responses of temperate trees to temperature increase and suggested this was evidence that warming has caused chilling and/or photoperiod to constrain phenology. We show that the apparent decrease in phenological sensitivity is mathematically expected under warming climates without any constraints from photoperiod or chilling. We substantiate the proposed mechanism using data from controlled conditions, simulations, and a long-term cherry blooming record series.

## Content

The effect of global warming on key life-cycle events of plant species is increasingly apparent, including widespread ground-based observations and satellite images that reveal significantly earlier phenological development in spring during the last decades^1^. However, recent studies have shown that the temperature sensitivity of spring phenological development has declined in temperate trees during the last two decades marked by substantial global warming^2–4^. This decreasing temperature sensitivity has often been attributed to changes in tree physiology, particularly to increasing limitation of chilling and photoperiodic cues. However, some authors also attributed adaptation to local climate as a driver of species-specific differences in temperature sensitivity^5,6^, while others demonstrated the importance of the duration of the period over which temperature is integrated for the interpretation of phenological sensitivity to temperature^7,8^.

Insufficient chilling or photoperiod limitations could affect the budburst or blooming of temperate species under certain environmental conditions^9^. Recent research, however, suggests chilling is usually fully satisfied in most temperate trees, and it would take far greater changes in chilling than currently observed to delay phenology^10^. Furthermore, experimental studies illustrate that the spring phenology of most northern woody plants is largely insensitive to changes in the length of the day with warming to date^5,11^. Taken together, these findings suggest that it may be premature to expect delayed budburst due to chilling and/or photoperiod constraints in most of the distribution of temperate trees, given current warming. Instead of a physiological change that delays the expected spring phenological advance with increasing temperature, the purely mathematical outcome of a simple degree-day model, with no chilling or photoperiod, may explain the progressive decline in phenological sensitivity with increasing temperature.

**Fig. 1:**
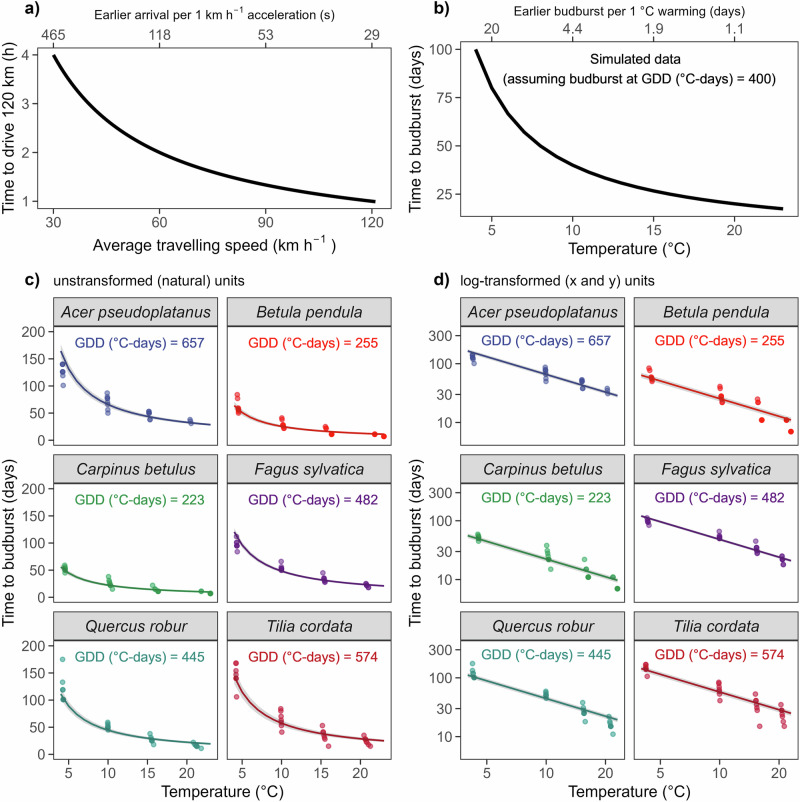
Non-linear responses to increasing temperatures. **a** The effect of car speed on the required time to drive a given distance. **b** The effect of temperature on the required time to accumulate a given thermal sum is fixed here as 400 GDDs. The time required to budburst decreases with increasing temperature exponentially, analogously to (**a**). **c** Time to budburst as a function of temperature under constant species-specific thermal sums. The solid line represents the modeled mean time to budburst, including the 0.95 confidence interval, as a function of forcing temperature. The dots represent the underlying raw data from twig cuttings exposed to different temperature conditions (*x*-axis) and a 16 h photoperiod until budburst after sufficient chilling conditions^5^. **d** The log–log transformed time to budburst-temperature relationship of the data shown in (**c**) to linearize the relationship.

The common thermal sum (or sum of degree days) model of budburst given daily temperatures is akin to modeling the time required to cover a given distance driving a car at a certain speed. In this analogy, daily average temperatures (‘speed’) and exposure time during spring integrate to fulfill the energy input required to budburst (Fig. 1a). As an analogy, if a car’s driving speed increases, the time to cover a given distance decreases non-linearly [see ref. ^12^]. Therefore, a person driving at low speed would save more time by driving 1 km h^−1^ faster than a person driving at a higher speed with the same speed increase (assuming the identical distance needs to be covered). Similarly, one would expect the required time for the spring phenological development of trees to decrease exponentially with increasing temperature, assuming that a species requires a fixed amount of accumulated heat to reach this event (Fig. 1b). Following this model, decreasing spring phenological sensitivity of tree species with increasing average temperature is mathematically expected. In support of this, models including the nonlinear temperature dependence of plant species have removed apparent declines in spring phenological sensitivity^13^.

We tested the effect of a species-specific fixed thermal sum as a model for deciduous tree spring phenology with experimental data from a climate chamber experiment to ensure no chilling or photoperiod limitation and with observational data from one of the five longest spring phenological time series [cherry (*Prunus avium*) blossom in Liestal, Switzerland^5,14^]. Tree cuttings from six European and three Chinese species were exposed to different chilling, photoperiod, and forcing temperature treatments in the original climate chamber experiment^5^. However, we included only data from European tree cuttings that experienced full chilling and were exposed to a photoperiod of 16 h to avoid potentially confounding effects of a lack of chilling or photoperiod on the temperature response of the twig cuttings.

Under fixed species-specific thermal sums (i.e., growing degree days, GDDs), the predicted non-linear relationship between time to budburst and temperature corresponds well with the observational data, which illustrates that temperature change could affect the apparent temperature sensitivity solely for mathematical reasons (Fig. 1c). Following the common mathematical transformation of such a threshold relationship, a log-log allows to linearize the exponential relationship between temperature and time to budburst and lead to effectively identical temperature sensitivities for all temperatures, in contrast to recent findings of declining sensitivities, which assumed a linear model (Fig. 1d).

A simulation of 500 random flowering dates based on 400 ± 50 GDD (mean ± standard deviation) and average spring temperatures of 6 ± 0.5 °C was run to show the non-linear relationship between average spring temperature and time to budburst under the assumption of constant GDD requirements (Fig. S1a). However, if the data of Fig. S1a are divided into several sections, each covering a temperature range of 3 °C, which is about the spring temperature range typically observed in long-term phenological time series [see ref. ^14^], a linear model represents the data of each section relatively well. Furthermore, the slopes of the linear models decrease when the temperature range of the 3 °C observation window increases (Fig. S1b–e), meaning that spring phenological temperature sensitivity changes based on the average temperature of the observational period without chilling and photoperiod constraints being involved.

**Fig. 2:**
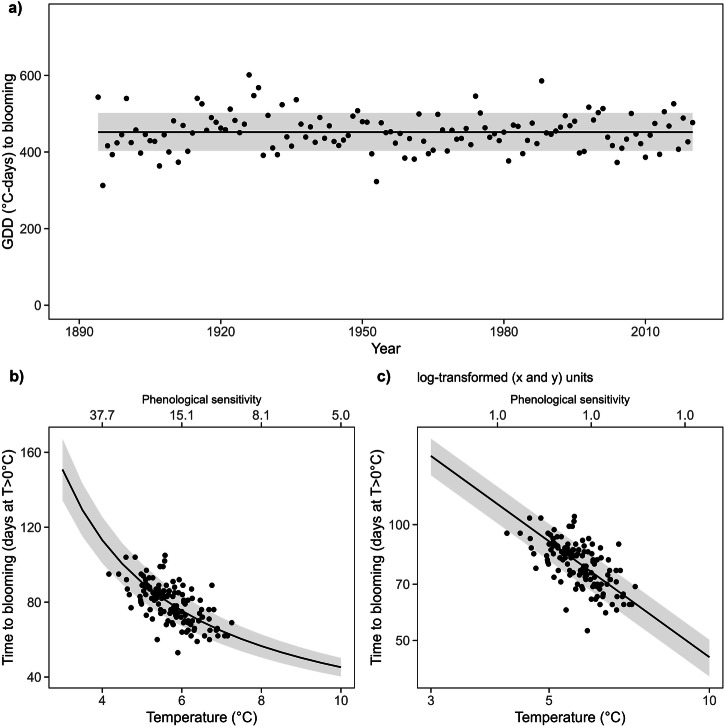
The apparent spring phenological sensitivity to temperature demonstrated in situ in one of the longest time series of phenological observations. **a** The growing degree days (GDD) required for cherry trees growing in Liestal to flower depend on the year. The regression line represents the long-term average GDD ±1*σ,* and the dots represent the underlying raw data of in situ phenological observations^14^. While we cannot rule out decreasing chilling as a driver of declining spring phenological temperature sensitivities, lower chilling should increase the required thermal time to budburst [see refs. ^5,10^], whereas here no systematic trend in GDD over time was observed. **b** Representation of the time to Liestal cherry tree blooming as a function of temperature under the assumption of constant GDD across years. The regression line represents simulated mean values ±1*σ*, and the dots represent the underlying raw data. **c** The log–log transformed time to blooming-temperature relationship of the data shown in (**b**) to linearize the relationship.

Under natural conditions, cherry trees (Liestal record) accumulated in-situ GDDs before blooming reveal some fluctuation between years but no long-term trend in thermal time required to budburst (Fig. 2a). A constant thermal sum requirement leads to a non-linear relationship between spring temperature and time to budburst, and thus, an apparent decrease in phenological sensitivity with increasing temperature (Fig. 2b). A log–log transformation again linearizes the expected non-linear relationship between temperature and time to budburst and leads to similar temperature sensitivities for all temperatures (Fig. 2c).

The outcome of those two case studies, including experimental and observational data, illustrates the importance of accounting for the non-linearity of the phenology response under warmer temperatures for future spring phenological predictions. Temperature sum concepts, such as GDDs, could thereby help to disentangle the mathematical effects from the physiological effects. Nevertheless, temperature sum concepts in ecology have some limitations and need to be interpreted carefully^7,8^.

We cannot rule out that physiological limitations, such as a lack of chilling or shorter photoperiod at the beginning of the vegetation period, may delay the spring phenological advance of temperate tree species in their native environment. Moreover, the proposed mathematical effect and tree physiological limitations could occur simultaneously in temperate forests and remain challenging to disentangle. Thus, the proposed mathematical model, including only forcing, predicts the observed declines, but was not included in previous reports^2,15^, suggesting it is important to additionally consider it in future work. Based on our results, we conclude that the recently observed decrease in phenological sensitivity under climate warming could partially be attributed to mathematical causes rather than the plant’s physiological response due to environmental constraints.

## Methodology

For our analysis, the mean species-specific heat accumulation sum (GDD) and the corresponding 0.95 confidence interval were calculated as a sum of daily average temperatures *T*_*i*_ across all days with a daily average temperature above a baseline temperature (*T*_base_) of 0 °C from the first day of temperature exposure *t*_1_ to budburst *t*_*BB*_ (see Eq. (1)).

1 $$GDD=\mathop{\sum }\limits_{{t}_{1}}^{{t}_{BB}}{T}_{i}$$

For the long-term time series analysis, GDD was calculated in a similar fashion (i.e., with 0 °C as *T*_base_) from January 1st to the blooming date of the Liestal cherry trees for each year from 1894 to 2020 separately, counting only days with average daily temperatures higher than 0 °C. for both GDD accumulation and calculation of the average temperature. The long-term average GDD ±1 standard deviation was divided by simulated temperatures ranging from 3 to 10 °C to predict the average expected number of days to budbreak, as well as the corresponding lower and upper levels of uncertainty. Thus, Fig. 2b represents simulated days to blooming from January 1st (i.e., DoY 1) under a given temperature and the underlying raw data for both parameters without days with average temperatures below 0 °C. Furthermore, alternative GDD requirements for Liestal cherries’ flowering were calculated with *T*_base_ of 2 °C and 5 °C, respectively, and then divided by the average temperature of all days above *T*_base_ between January 1st and the flowering date to substantiate the principle (Fig. S2).

Spring phenological sensitivity (*S*_*T*_) was calculated for 1 °C temperature intervals from a given temperature *T*_*i*_ to the temperature ${T}_{i+{1}\,^{\circ }{{C}}}$ and the corresponding time to blooming ${t}_{{T}_{i}}$ and ${t}_{{T}_{i+{1}\,^{\circ }{{C}}}}$. In detail, Eq. (2) was used for untransformed (natural) units, and Eq. (3) was used for log-transformed (x and y) units.

2 $${S}_{T}=\frac{{t}_{{T}_{i}}-{t}_{{T}_{i+{1}\,^{\circ }{{C}}}}}{| {T}_{i}-{T}_{i+{1}^{\circ }C}| }$$

3 $${S}_{T}=\frac{{\rm{log}}({t}_{{T}_{i}})-{\rm{log}}({t}_{{T}_{i+{1}\,^{\circ }{{C}}}})}{| {\rm{log}}({T}_{i})-{\rm{log}}({T}_{i+{1}\,^{\circ }{{C}}})| }$$

## Supplementary information

Supplementary Information

## Data Availability

No datasets were generated during the current study.

## Code availability

The authors will make the code available upon request in the case of publication.
