# Supplementary material for: Rethinking the interpretation of spring phenological temperature sensitivity
Source: NPJ Sci Plants. 2025 Aug 4;1(1):3. doi: 10.1038/s44383-025-00004-6 (PMC12321564; doi:10.1038/s44383-025-00004-6)
Supplement: Supplementary file 1 — Supplementary Information [file 44383_2025_4_MOESM1_ESM.pdf]

Supplementary information

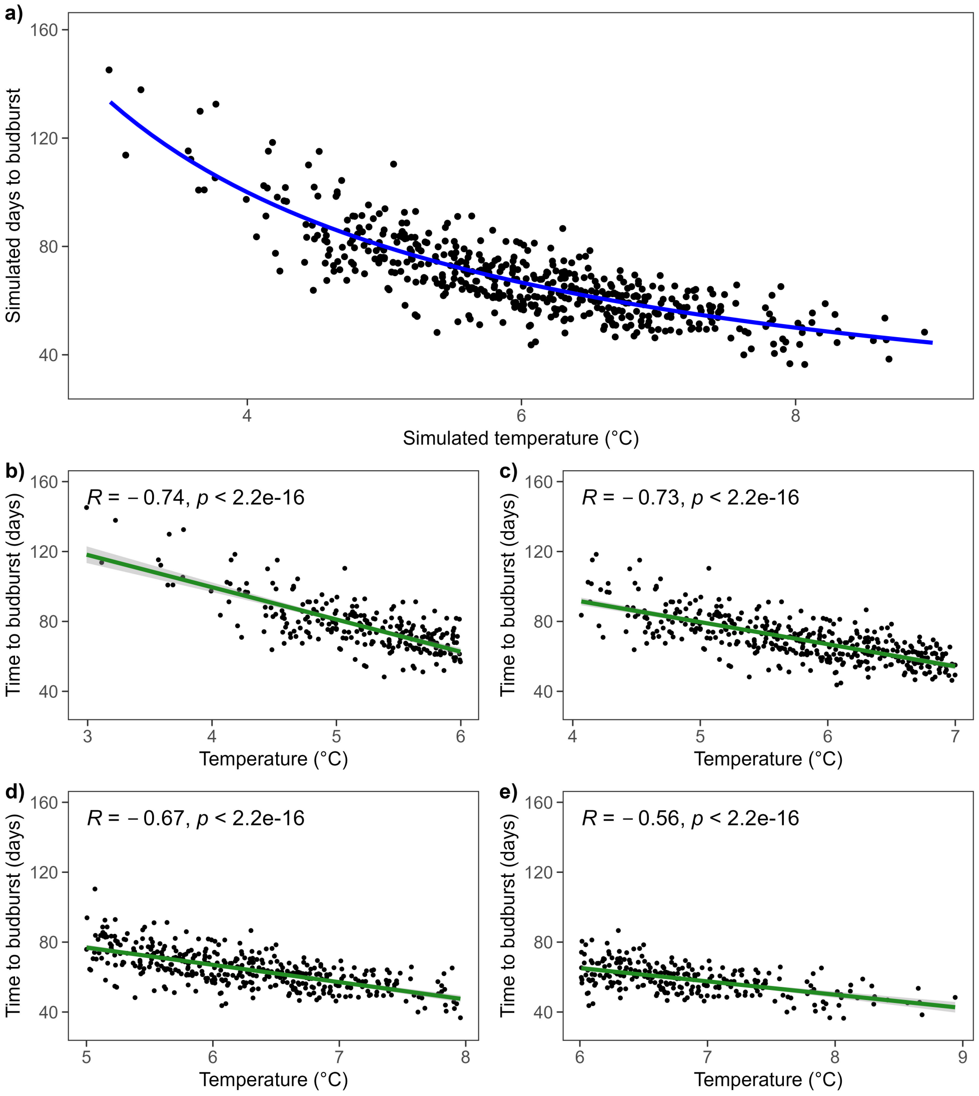

Fig. S1 Illustration of required days to budburst based on simulated GDD (mean = 400, standard deviation = 50) and spring temperatures in °C (mean = 6, standard deviation = 0.5). The uppermost panel shows the underlying hypothetical relationship with a blue line covering average spring temperatures from 3 to 9°C and black dots representing simulated data (a). The lower panels show that linear models, illustrated in green represent the temperature-time to budburst relationship well, as long as only temperature windows of 3°C are considered (b, c, d, and e). The slopes of the linear models decrease from panel b to panel e, meaning that spring phenological temperature sensitivity changes based on the average temperature of the observational period without chilling and photoperiod constraints being involved. The black dots of the lower four panels represent subsets of the simulated data used in panel a.

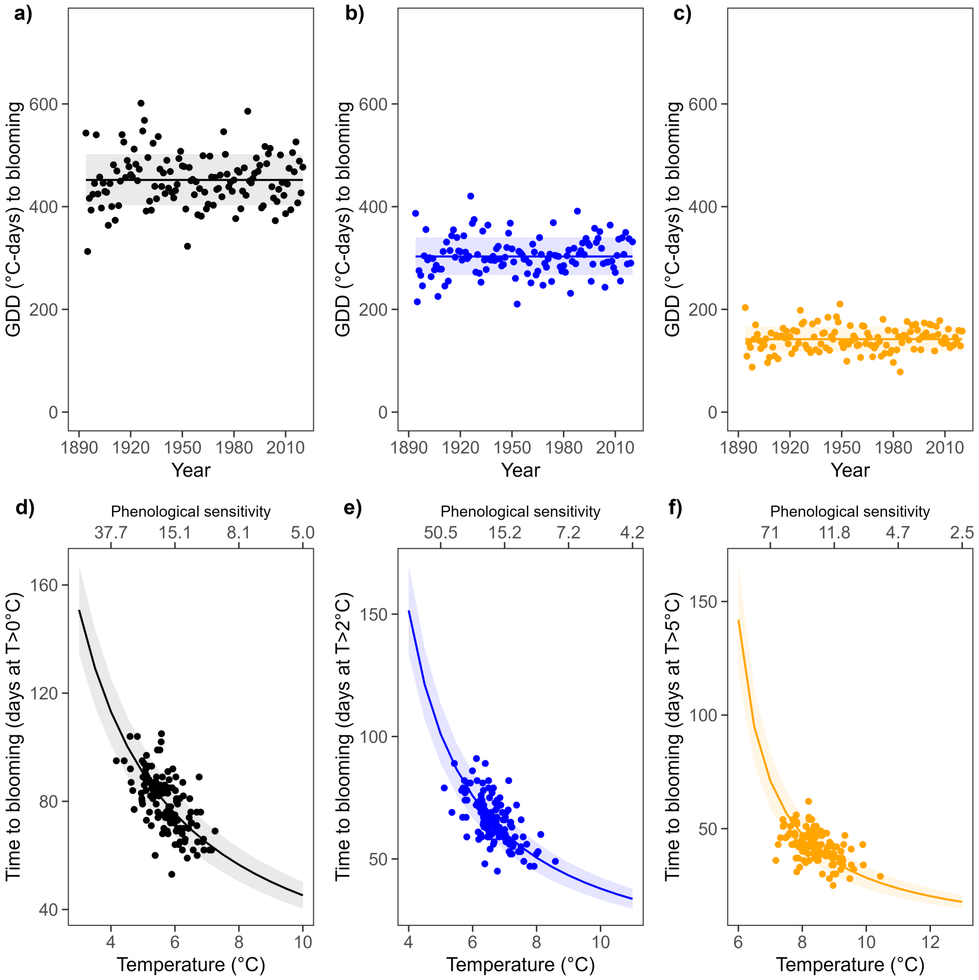

Fig. S2 The growing degree days GDD required for cherry trees growing in Liestal to flower dependent on the year if baseline temperatures (T_{base}) of 0°C (a), 2°C (b), or 5°C are used (c). The regression line represents long-term average GDD ± 1 standard deviation and the dots represent the underlying raw data of in-situ phenological observations (Vitasse et al, 2022). While we cannot rule out decreasing chilling as a driver of declining spring phenological temperature sensitivities, lower chilling should increase the required thermal time to budburst (see Baumgarten et al, 2021; Walde et al, 2022), whereas here no systematic trend in GDD over time was observed. Representation of the time to Liestal cherry tree flowering as a function of temperature under the assumption of constant GDD across years if T_{base} of 0°C (d), 2°C (e), or 5°C are used (f). The regression line represents simulated means ± 1 standard deviation and the dots represent and the underlying raw data
